# Workplace violence, work‐related exhaustion, and workplace cognitive failure among nurses: A cross‐sectional study

**DOI:** 10.1111/jan.16217

**Published:** 2024-05-07

**Authors:** Judith E. Arnetz, Nathan Baker, Eamonn Arble, Bengt B. Arnetz

**Affiliations:** ^1^ Department of Family Medicine, College of Human Medicine Michigan State University Grand Rapids Michigan USA; ^2^ Department of Psychology Michigan State University East Lansing Michigan USA; ^3^ Department of Psychology Eastern Michigan University Ypsilanti Michigan USA

**Keywords:** nursing, structural equation modelling, workplace cognitive failure, workplace efficiency, workplace violence

## Abstract

**Aim:**

To examine the relationships between nurses' exposure to workplace violence and self‐reports of workplace cognitive failure.

**Design:**

A cross‐sectional study.

**Methods:**

An online questionnaire was administered in April 2023 to nurses in Michigan, US. Structural equation modelling was used to examine effects of physical and non‐physical workplace violence (occupational stressors) and work efficiency and competence development (occupational protective factors) on workplace cognitive failure.

**Results:**

Physical violence was a significant predictor of the action subscale of cognitive failure. There were no direct effects of non‐physical violence, workplace efficiency, or competence development on any of the workplace cognitive failure dimensions. Both types of violence and efficiency had significant indirect effects on workplace cognitive failure via work‐related exhaustion. Work‐related exhaustion predicted significantly higher scores for workplace cognitive failure.

**Conclusion:**

Workplace violence and work efficiency exhibited primarily indirect effects on workplace cognitive failure among nurses via work‐related exhaustion.

**Implications for the Profession and/or Patient Care:**

Nurses experiencing workplace violence may be at increased risk for workplace cognitive failure, especially if they are also experiencing work‐related exhaustion. Workplaces that nurses perceive as more efficient can help to mitigate the effects of violence on nurses' cognitive failure.

**Impact:**

This study addressed the possible effects of workplace violence as well as work efficiency and competence development on nurses' cognitive failure at work. Analyses revealed primarily indirect effects of workplace violence, and indirect protective effects of work efficiency, on nurses' cognitive failure via work‐related exhaustion. This research has implications for healthcare organizations and suggests that efforts made by healthcare workplaces to prevent violence and work‐related exhaustion, and to enhance work efficiency, may help to mitigate workplace cognitive failure among nurses.

**Reporting Method:**

We have followed the STROBE checklist in reporting this study.

**Patient or Public Contribution:**

No Patient or public contribution.

## 1 INTRODUCTION

Workplace violence is a recognized occupational hazard in the healthcare sector (Liu et al., 2019; Rossi et al., 2023), with nurses at increased risk for both violent events (Lozano et al., 2021; Rossi et al., 2023) and violence‐related injury (Groenewold et al., 2018) compared to other healthcare providers. A growing number of studies provide evidence of an increase in violence against nurses during the COVID‐19 pandemic (Byon et al., 2022; Chirico et al., 2022; McGuire et al., 2022), and in its aftermath (Brigo et al., 2022). In a U.S. national survey, 48% of nurses reported experiencing a significant increase in workplace violence since 2021 (National Nurses United, 2022). Violence towards nurses is most often perpetrated by patients or their family members, (McPhaul & Lipscomb, 2004), although violence from colleagues and other employees, including bullying (Arnetz, Fitzpatrick, et al., 2019; Arnetz, Sudan, et al., 2019; Bambi et al., 2018), is also prevalent (Arnetz, Aranyos, et al., 2011). Violence towards nurses is a global problem (Kafle et al., 2022; Varghese et al., 2022; Zhao, Xie, et al., 2018) that has been associated with increased anxiety, depression (Zhao, Xie, et al., 2018), turnover, and burnout (Duan et al., 2019; Laeeque et al., 2018; Zhao, Shi, et al., 2018) among victims. Nurses react to violent events with anger, sadness, disappointment, fear, and an increased tendency to be on their guard in patient interactions (Arnetz & Arnetz, 2001). Violent events also present nurses with a conundrum, as being assaulted or threatened by the person they are caring for professionally results in cognitive dissonance (Privitera & Arnetz, 2011).

### 1.1 Workplace violence and cognitive failure

Nurses exposed to workplace violence have reported feeling the effects of violent events long after they occur (Lanctot & Guay, 2014), even if the event did not result in serious physical injury (Gates et al., 2011). Studies have examined the impact of workplace violence on nurses' work performance (Atan et al., 2012; Fischer & Martinez, 2013; Gates et al., 2011; Magnavita et al., 2020; Samir et al., 2012), reporting a negative effect on nurse behaviour (Samir et al., 2012) or a decrease in work productivity (Atan et al., 2012) or work ability (Fischer & Martinez, 2013). However, the negative effects or the mechanisms behind them are not clearly defined. An Italian study found significantly worse work functioning among nurses exposed to workplace violence compared to non‐exposed. Nurses who experienced violence scored higher on conflicts with colleagues, impaired contact with patients/family members, and lack of energy/motivation, but there were no differences between groups on execution of cognitive tasks (Magnavita et al., 2020). However, a U.S. study of emergency department nurses found that nurses experiencing workplace violence had increased difficulty in staying cognitively focused on their work, resulting in diminished productivity. Nurses reported difficulty keeping their mind on their work, thinking clearly, and concentrating (Gates et al., 2011). The authors suggested that the experience of violence at work is traumatizing and negatively impacts nurses' ability to cognitively focus, although most nurses reported being able to continue working. A recent Chinese study also reported greater perceived cognitive deficits among nurse victims of violence compared to non‐victims (Ding et al., 2023). A systematic review examined the non‐somatic effects of patient aggression on nurses and found that these included cognitive changes such as impaired concentration (Needham et al., 2005). These studies indicate that workplace violence can negatively affect nurses' ability to meet cognitive demands, potentially impacting their ability to carry out patient care safely. However, they did not specifically measure work‐related cognitive failures. Linking violence to work‐related cognitive failures would inform workplace interventions to help nurses counteract the cognitive effects of violence exposure.

## 2 BACKGROUND

### 2.1 Workplace cognitive failure among nurses

Cognitive failures are defined as lapses in perception, memory, and actions during tasks that an individual would normally be able to carry out (Broadbent et al., 1982). The Workplace Cognitive Failure Scale (WCFS), a validated measure specifically related to work tasks, was developed with a focus on the implications of such failures for work safety behaviours and outcomes. The 15‐item scale comprises three subscales that assess the frequency of experiencing lapses in memory, attention, or physical action, respectively (Wallace & Chen, 2005). In nurses, workplace cognitive failure has been associated with task‐related stressors (Elfering et al., 2011), work‐flow interruptions (Elfering et al., 2015), work stress (Kakemam et al., 2019) and adverse patient events (Kakemam et al., 2019; Park & Kim, 2013). Cognitive failure is a critical area of study in the context of nursing work processes because nursing work demands a high level of cognitive functioning that has been associated with prevention of errors that may jeopardize patient care (Elfering et al., 2011). A study of critical care nurses underscored the significance of cognitive “vigilance and alertness” in nurses' ability to discover and correct potential medication, procedural, and documentation errors (Rogers et al., 2008, p.119). When nurses' ability to perform their duties decreases due to a high workload and job stress, their mental resources are taxed, which may impair their perception, memory, and ability to perform important cognitive tasks (Abbasi et al., 2016). Building on prior research suggesting that workplace violence may impair nurses' cognitive ability (Ding et al., 2023; Gates et al., 2011; Needham et al., 2005), the current study focuses on the association between workplace violence and workplace cognitive failure and the potential protective effects of other work‐related factors.

### 2.2 Conceptual model

**FIGURE 1:**
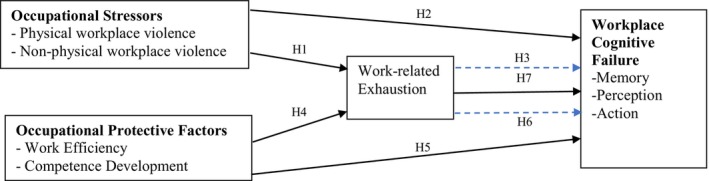
Conceptual model of hypothesized relationships between occupational stressors and protective factors, work‐related exhaustion, and workplace cognitive failure. Dotted lines indicate indirect effects.

To better understand the potential effects of workplace violence on nurses' workplace cognitive failure, we developed a conceptual model based on the Conservation of Resources (COR) theory (Hobfoll, 1989). COR posits that individuals strive to gain, maintain, and enhance personal resources to withstand adversity and thrive. Thriving results in resource gain, while inability to thrive results in resource loss, making it increasingly difficult for an individual to cope with environmental stressors and creating “loss spirals” (Hobfoll, 1989; Viotti, Converso, et al., 2018). This model has proven useful among nurses (Arnetz et al., 2021; Prapanjaroensis et al., 2017; Viotti, Converso, et al., 2018; Viotti, Essenmacher, et al., 2018). As illustrated in Figure 1, we hypothesized that occupational stressors could increase workplace cognitive failure (resource loss) while occupational protective factors could reduce cognitive failure (resource gain).

#### 2.2.1 Occupational stressors

Prior studies indicate that occupational stressors can influence cognitive failure in nurses (Arble et al., 2023; Arnetz et al., 2021). During the early stages of the COVID‐19 pandemic, a questionnaire study of 695 U.S. nurses found that 75% endorsed some degree of workplace cognitive failure, which was associated with frequent contact with COVID patients and lack of personal protective equipment. The effects of those physical exposures to COVID‐19 on cognitive failure were mediated by work stress and work‐related exhaustion (Arnetz et al., 2021). Increased substance use among nurses during the pandemic was also associated with workplace cognitive failure via mediating effects of anxiety, posttraumatic stress disorder, and secondary trauma (Arble et al., 2023). Building on the research that workplace violence may impair nurses' cognitive ability (Ding et al., 2023; Gates et al., 2011; Needham et al., 2005), occupational stressors in the current study were operationalized as physical and non‐physical workplace violence. Both forms of violence have been shown to impact nurse health and well‐being (Gerberich et al., 2004; Lanctot & Guay, 2014; Needham et al., 2005), and several studies have indicated that the negative health effects of non‐physical violence may be even greater than those of physical violence (Gerberich et al., 2004; Needham et al., 2005). It was therefore interesting to examine each of these stressors independently for their association to workplace cognitive failure.

#### 2.2.2 Occupational protective factors

Research has identified occupational protective factors, including a positive work environment and coworker support, that can counteract the negative effects of workplace violence on nurses, such as burnout (Lozano et al., 2021). However, the literature on workplace cognitive failure in healthcare focuses on risk factors, e.g., work stress and task interruptions (Elfering et al., 2011, 2014, 2015), with a lack of studies on protective factors. In the current study, we examined work efficiency and competence development as workplace factors that could potentially protect against workplace cognitive failure. *Work efficiency*, a measure of employees' perceptions of how well work processes function at their workplace (Arnetz, Lucas, & Arnetz, 2011; Arnetz, Zhdanova, et al., 2011), has been shown to have a protective effect (resource gain) on both incivility (Viotti, Converso, et al., 2018; Viotti, Essenmacher, et al., 2018) and physical violence (Arnetz et al., 2018) against healthcare workers. A study of nursing work characteristics and cognitive failure reported that frequent interruptions at work can cause nurses to be distracted from the task at hand, leading to an increased need to increase their working memory load (Elfering et al., 2011). A workplace characterized by high efficiency would, by definition, experience fewer interruptions and could therefore potentially have a protective effect on workplace cognitive failure (Elfering et al., 2015).

*Competence development* measures employees' perceptions of opportunities to utilize and develop their professional skills at work (Arnetz & Hasson, 2007). In a study of hospital nurses, competence development opportunities at work had a protective effect (resource gain) on nurse disengagement due to coworker bullying (Arnetz, Sudan, et al., 2019). In qualitative studies, nurses reported that bullying at work resulted in decreased opportunities for competence development, i.e., resource loss (Arnetz, Fitzpatrick, et al., 2019; Hutchinson et al., 2010). In a longitudinal study, cognitive stimulation at work was associated with improved cognitive functioning in both young and older workers. Cognitive stimulation was measured as training opportunities at work, the cognitive “richness” of work content, and the degree of cognitive effort that the work entailed (Marquie et al., 2010). Nursing work is complex and cognitively demanding (Jackson et al., 2021). We therefore posited that nurses with opportunities for competence development at work would have greater cognitive stimulation that could potentially protect against workplace cognitive failure.

#### 2.2.3 Work‐related exhaustion as a mediator

*Work‐related exhaustion* is a measure of employees' feelings of mental and physical fatigue related to their work (Arnetz & Hasson, 2007). Akin to burnout, work‐related exhaustion has also been linked with cognitive failure in nurses, mediating the relationship between COVID‐19 exposure and cognitive failure (Arnetz et al., 2021). Considering the significant increase in burnout in nurses since the onset of the COVID‐19 pandemic (Aiken et al., 2023; Nigam et al., 2023), it was especially interesting to examine the role of work‐related exhaustion in violence exposure and workplace cognitive failure. In previous research, workplace incivility (Viotti, Essenmacher, et al., 2018) and physical violence (Duan et al., 2019) were associated with increased work‐related exhaustion (resource loss). Magnavita et al. (2020) found that nurses exposed to workplace violence experienced a greater lack of energy (resource loss) than non‐exposed nurses. In a systematic review of the non‐somatic effects of patient aggression on nurses, Needham et al. (2005) found four main effects: biophysiological, emotional, cognitive, and social. The biophysiological response was decreased energy and hyperarousal, while the emotional response included feelings of burnout, confusion, and numbness. We posit that these nurse reactions reflect work‐related exhaustion that could play a central role in influencing workplace cognitive failure. Thus, exhaustion could act to mediate the proposed relationship between violence and workplace cognitive failure.

## 3 THE STUDY

Considering workplace violence as a chronic stressor in the nursing profession that is increasing, the current study aimed to examine the relationships between nurses' exposure to workplace violence and self‐reports of workplace cognitive failure. Operationalizing occupational stressors as violent events, we hypothesized that both physical and non‐physical violence would be positively associated with work‐related exhaustion (H1). Furthermore, violence at work would be positively associated with workplace cognitive failure directly (H2) and indirectly through work‐related exhaustion (H3).

In addition to the potential negative effects of stressful work events (i.e., violent events) on cognition, we further hypothesized that positive occupational factors – specifically, work efficiency and competence development – would have a protective effect on work‐related exhaustion (H4). We also hypothesized direct effects of work efficiency and competence development on workplace cognitive failure (H5) and indirect effects through work‐related exhaustion (H6). Finally, we hypothesized direct positive effects of work‐related exhaustion on workplace cognitive failure (H7). These hypotheses are depicted in Figure 1.

## 4 METHODS

### 4.1 Study design

A cross‐sectional online survey was conducted in a sample of nurses in Michigan, U.S., in April 2023.

### 4.2 Participants

Participants were registered nurses (RN), advanced practice registered nurses (APRN) and licensed practical nurses (LPN) who were members of the American Nurses Association (ANA) Michigan, the Michigan Organization of Nurse Leaders (MONL), and the Coalition of Michigan Organizations of Nursing (COMON). All members of the three organizations, totaling approximately 18,300 and representing 18% of Michigan working nurses, were eligible to participate. Members represent all practice disciplines and work settings, including hospitals, primary care, community‐based facilities, and nursing homes across the state.

### 4.3 Data collection

Each organization distributed an invitation by email to its organization's members. The invitation explained that the survey was anonymous and confidential. Those nurses agreeing to participate completed a consent statement in Qualtrics before responding to the survey. This method was utilized in a previous study in May 2020 (Arble et al., 2023; Arnetz et al., 2021; Arnetz, Goetz, Arnetz, et al., 2020; Arnetz, Goetz, Sudan, et al., 2020).

### 4.4 Study variables

The online survey was comprised of 88 items measuring personal and work‐related demographics, history of COVID‐19 illness, mental health, general health and well‐being, lifestyle, and occupational experiences including work‐related exhaustion, workplace cognitive failure, workplace violence, work efficiency, and competence development.

### 4.5 Independent variables

Individual demographic variables included age, gender identity, race/ethnicity, and history of testing positive for COVID‐19 (response alternatives no; yes, once; yes, more than once). Work‐related demographics included profession and practice setting.

Occupational stressors were operationalized as nurses' reports of *physical and non‐physical workplace violence*, respectively. Two categorical items asked nurses whether they had been a target of physical violence (such as hitting, spitting, scratching, kicking) and/or non‐physical violence (such as yelling, cursing, threatening, bullying, verbal aggression) in the past year. Response alternatives were “no, never”; “yes, once or twice”; and “yes, several times.” For each type of violence, respondents were asked who the perpetrator was, whether they sustained any physical injury, and whether they lost any time away from work as a result of the violent incident.

Occupational protective factors were operationalized by two subscales from the Quality Work Competence (QWC) questionnaire, an organizational climate survey that measures employees' perceptions of their work environment (Arnetz, 1997; Arnetz & Hasson, 2007). QWC has shown excellent psychometric properties in numerous studies (e.g., Arnetz, 1997; Arnetz & Hasson, 2007; Arnetz, Lucas, & Arnetz, 2011; Arnetz, Zhdanova, et al., 2011) and has been validated using biomarkers of stress (Anderzen & Arnetz, 2005; Arnetz, Sudan, et al., 2019). *Workplace efficiency* is a 4‐item scale measuring employees' perception of how efficiently their workplace operates. Response alternatives range from (1) strongly disagree to (4) strongly agree. A sample item is, “At my workplace, resources are optimally utilized.” This measure has demonstrated adequate reliability and validity in previous studies (Anderzen & Arnetz, 2005; Arnetz, Lucas, & Arnetz, 2011; Arnetz, Zhdanova, et al., 2011), with alpha values exceeding 0.75 and strong concurrent validity with independent measures of efficiency (Anderzen & Arnetz, 2005). *Competence development* is a 4‐item scale measuring the degree to which individuals develop professionally and can utilize their competence in their work. Response alternatives are on a four‐point scale ranging from (1) never to (4) often. A sample item is, “Do you develop professionally in your work?” The measure has demonstrated adequate reliability and validity in previous studies (Arnetz & Hasson, 2007; Hasson & Arnetz, 2008) with alpha values exceeding 0.76 and concurrent validity with independent measures of skills' development (Arnetz & Hasson, 2007). In this study, Cronbach's alpha was 0.85 for workplace efficiency and 0.89 for competence development.

*Work‐related exhaustion*, a 3‐item QWC subscale (Arnetz, 1997; Arnetz & Hasson, 2007), was included as a possible mediator of the effects of occupational factors on workplace cognitive failure. The subscale asks respondents to rate how often they feel emotionally drained after work, worn out after work, and tired when they think about work. Response alternatives are on a five‐point scale ranging from (1) never to (5) daily. The measure has demonstrated good reliability and validity in previous studies, with alphas of 0.84 or higher (Arnetz & Hasson, 2007; Hasson & Arnetz, 2008). Cronbach's alpha for Work‐related exhaustion in this study was 0.93.

Responses for each QWC subscale were summed to a total score, with higher scores indicating greater competence development, workplace efficiency, and work‐related exhaustion/burnout.

### 4.6 Dependent variables

The subscales of the Workplace Cognitive Failure Scale (WCFS, Wallace & Chen, 2005) were the main outcome variables. The WCFS is a 15‐item scale that assesses the frequency with which employees experience cognitive lapses while working. The WCFS has demonstrated adequate construct and criterion validity with employee‐reported safety outcomes as well as objective measures of work injury and supervisor ratings of employee safety behaviour (Wallace & Chen, 2005). The instrument has also demonstrated good validity and reliability in studies conducted in Switzerland (Elfering et al., 2011, 2014, 2015) and Iran (Kakemam et al., 2019), with alphas ranging from 0.77–0.92. The scale is comprised of three 5‐item subscales measuring lapses in memory, attention, and action, respectively. All items are rated on a 5‐point response scale ranging from (1) never to (5) very often. A sample item from the memory subscale is, “Fail to recall work procedures.” For the attention subscale, a sample item is, “Do not focus your full attention on work activities.” A sample item from the Action subscale is, “Throw away something you meant to keep (e.g., memos, tools).” Scores for each subscale are calculated by summing the component items, with higher values indicating greater levels of cognitive failure. Cronbach's alpha for the subscales in the current study were .86 for memory, .86 for attention, and .82 for action.

### 4.7 Data analysis

Descriptive statistics were used to summarize individual and work demographics of study participants. Bivariate analyses using Pearson's *r* were used to assess the correlations between the continuous study variables Workplace efficiency, Competence development, Work‐related exhaustion, and the WCFS subscales Memory, Attention, and Action. Statistical analyses were conducted using IBM SPSS statistics, V.28, 2021 (IBM Corp, Armonk, NY). A two‐sided *p*‐value <.05 was deemed statistically significant. To test study hypotheses, structural equation modelling using maximum likelihood estimation was conducted using Mplus 7 (Muthén & Muthén, 2012). Direct effects of occupational stressors (physical and non‐physical workplace violence) and occupational protective factors (work efficiency, competence development) on WCFS subscales were examined, as were indirect effects via work‐related exhaustion. Model fit was examined using multiple fit indices including the chi‐square of the model, comparative fit index (CFI); root mean square error of approximation (RMSEA); and standardized root mean squared residual (SRMR). We followed commonly accepted guidelines for evaluating these fit indices, deeming CFI > 0.90, and RMSEA and SRMR <0.08 to indicate acceptable model fit (Kenny, 2020; Shi et al., 2019). Confidence intervals for direct effects within the model were estimated using a symmetric approach based on the standard error for each effect, while indirect effects and confidence intervals were estimated using bias‐corrected bootstrapping with 1000 sample draws (Muthén & Muthén, 2012).

### 4.8 Power analysis

The data were generated using convenience sampling. An overall test of the structural equation model's power to detect model misfit was conducted using MacCallum et al.'s (2006) approach to detect model power. Analyses performed using Preacher & Coffman's (2006) syntax indicated that the sample size was sufficient to detect small differences in the model fit with 96% power. To estimate the power to detect significance in the path model, we selected the path with the most predictors and estimated the power to detect significant effects using G*Power 3.1 (Faul et al., 2009). This analysis suggested our sample was sufficient to provide 95% power to detect small effects (*f* ^2^ = 0.03, α = .05).

### 4.9 Ethical considerations

The study was approved by the Institutional Review Board at Michigan State University (Study ID 00004459).

## 5 RESULTS

### 5.1 Characteristics of study participants

A total of 505 nurses responded to the survey, representing a response rate of approximately 3% of all eligible nurses. Characteristics of study participants are summarized in Table 1. The sample was majority female (91%, *n* = 460), Caucasian (81%, 411), and younger than 45 years (58%, *n* = 293). The sample was similar to the total population of Michigan nurses in terms of gender and ethnicity (MPHI, 2022). The majority of respondents were registered nurses (92%, *n* = 474), and worked in adult medical/surgical contexts (40%, *n* = 195), followed by intensive care (12%, *n* = 61), obstetrics/labor and delivery (12%, *n* = 61), and emergency (10%, *n* = 53). The rest of the sample worked in a variety of practice settings including mental health, paediatrics, primary/ambulatory care, and hospice/home care/long‐term care. Only 1% (*n* = 7) of the sample was unemployed or retired.

**TABLE 1:** Characteristics of study participants (*n* = 505).

	*N* (%)^b^
Age (years)	
18–24	27 (5.3)
25–34	158 (31.3)
35–44	108 (21.4)
45–54	107 (21.2)
55–64	75 (14.9)
65–74	20 (4.0)
75+	6 (1.2)
Prefer not to answer	4 (0.8)
Gender identity	
Males	40 (7.9)
Females	460 (91.1)
Other	1 (0.2)
Prefer not to answer	4 (1.0)
Race	
White	411 (81.4)
Black/African American	37 (7.3)
Other	57 (11.4)
Hispanic origin	
Yes	18 (3.6)
No	474 (93.9)
Prefer not to answer	
Profession	
Advanced practice registered nurse (APRN)	13 (2.6)
Licensed practical nurse	28 (5.5)
Registered nurse	464 (91.9)
Practice setting	
Adult Medical/Surgical	195 (39.6)
Emergency	53 (10.5)
Intensive care	61 (12.1)
Mental health	12 (2.4)
Paediatrics	30 (5.9)
Primary care/ambulatory care	11 (2.2)
Obstetrics/labor & delivery	61 (12.1)
Hospice, home care, long‐term care	9 (1.8)
Ever tested positive for COVID‐19	
No	119 (24%)
Yes, once	246 (49%)
Yes, more than once	126 (25%)

^b^ Numbers do not add to group totals due to missing values.

### 5.2 Nurses' reports of workplace violence

Table 2 presents nurses' reported experiences with workplace violence during the past year. Nearly 40% (39.3%, *n* = 172) reported being targets of physical violence, while 63.7% (*n* = 277) were targets of non‐physical violence. Nurses reported that patients were the primary source of both physical (32.7%, *n* = 165) and non‐physical violence (42.2%, *n* = 213). For non‐physical violence, nurses also reported patient relatives/visitors (26.1%, *n* = 132) and fellow employees (10.3%, *n* = 52) as perpetrators of violence. The majority of nurses (95%, *n* = 409) did not sustain physical injuries or lose time away from work (99.3%, *n* = 431) due to violence at work.

**TABLE 2:** Experience with workplace violence in the past year (*n* = 505).

	*N* (%)^a^
Target of *physical* violence	
No	266 (60.7)
Yes, once or twice	123 (28.1)
Yes, several times	49 (11.2)
Perpetrator of *physical* violence	
Patient	165 (32.7)
Patient relative/visitor	32 (6.3)
Employee or co‐worker	4 (0.8)
Supervisor/manager	1 (0.2)
Other	3 (0.6)
Target of *non‐physical* violence	
No	158 (36.3)
Yes, once or twice	156 (35.9)
Yes, several times	121 (27.8)
Perpetrator of *non‐physical* violence	
Patient	213 (42.2)
Patient relative/visitor	132 (26.1)
Employee or co‐worker	52 (10.3)
Supervisor/manager	11 (2.2)
Other	10 (2.0)
Physical injury from a violent incident	
No	409 (94.7)
Yes	23 (5.3)
Sustained injury to head or face	
No	19 (82.6)
Yes	4 (17.4)
Lost time away from work from a violent incident	
No	431 (99.3)
Yes	3 (0.7)

^a^ Multiple responses were possible.

### 5.3 Nurse self‐reported workplace cognitive failure

Some degree of workplace cognitive failure (responses of sometimes, often, or very often) was endorsed by 74% of nurses. Nurses who were unemployed or retired were instructed to skip these questions on the survey. Descriptive statistics and bivariate correlations between the continuous study variables, the total WCFS and its three subscales are shown in Table 3. The mean score for total WCFS was 26.90 (*SD =* 9.13). For the three subscales, mean scores were highest for Attention (9.90, *SD* = 3.87) and Memory (9.12, *SD* = 3.57) and lowest for Action (7.89, *SD* = 2.83). Work‐relation exhaustion had a mean score of 9.67 (*SD =* 3.32), workplace efficiency had a mean of 12.03 (*SD =* 2.21), and competence development had a mean of 13.84 (*SD =* 2.38). WCFS and its corresponding subdimensions all had significant negative correlations with workplace efficiency (*r* ranging from *r* = −.14 to −.17) and competence development (*r* ranging from −.12 to −.15). Work‐related exhaustion was instead positively correlated with all dimensions of WCFS (*r* = .35 for total WCFS).

**TABLE 3:** Bivariate correlations (Pearson's *r*) between, workplace efficiency, competence development, work‐related exhaustion, and cognitive failure symptoms (*n* = 430–445).

Variable (score range)	Mean (SD)	1	2	3	4	5	6	7
1 Workplace efficiency (4–16)	12.03 (2.21)	**.85**						
2 Competence Development (4–16)	13.83 (2.38)	.48***	**.89**					
3 Work‐related exhaustion (3–15)	9.67 (3.32)	−.26***	−.20***	**.93**				
4 WCFS (15–75)	26.90 (9.13)	−.17***	−.14***	.35***	**.93**			
5 Memory – WCFS (5–25)	9.12 (3.57)	−.17***	−.15***	.33***	.90***	**.86**		
6 Attention – WCFS (5–25)	9.90 (3.87)	−.15**	−.11**	.34***	.90***	.71***	**.86**	
7 Action – WCFS (5–25)	7.89 (2.83)	−.14**	−.12**	.27***	.86***	.69***	.66***	**.82**

*Note*: Variable score ranges, means, and standard deviations (SD) are reported. WCFS, Workplace cognitive failure scale; higher values on WCFS and each subscale indicate more symptoms of cognitive failure. Cronbach's alpha reported in bold for each scale along the diagonal.

** *p* < .01;

*** *p* < .001.

### 5.4 Work‐related factors associated with workplace cognitive failure

**FIGURE 2:**
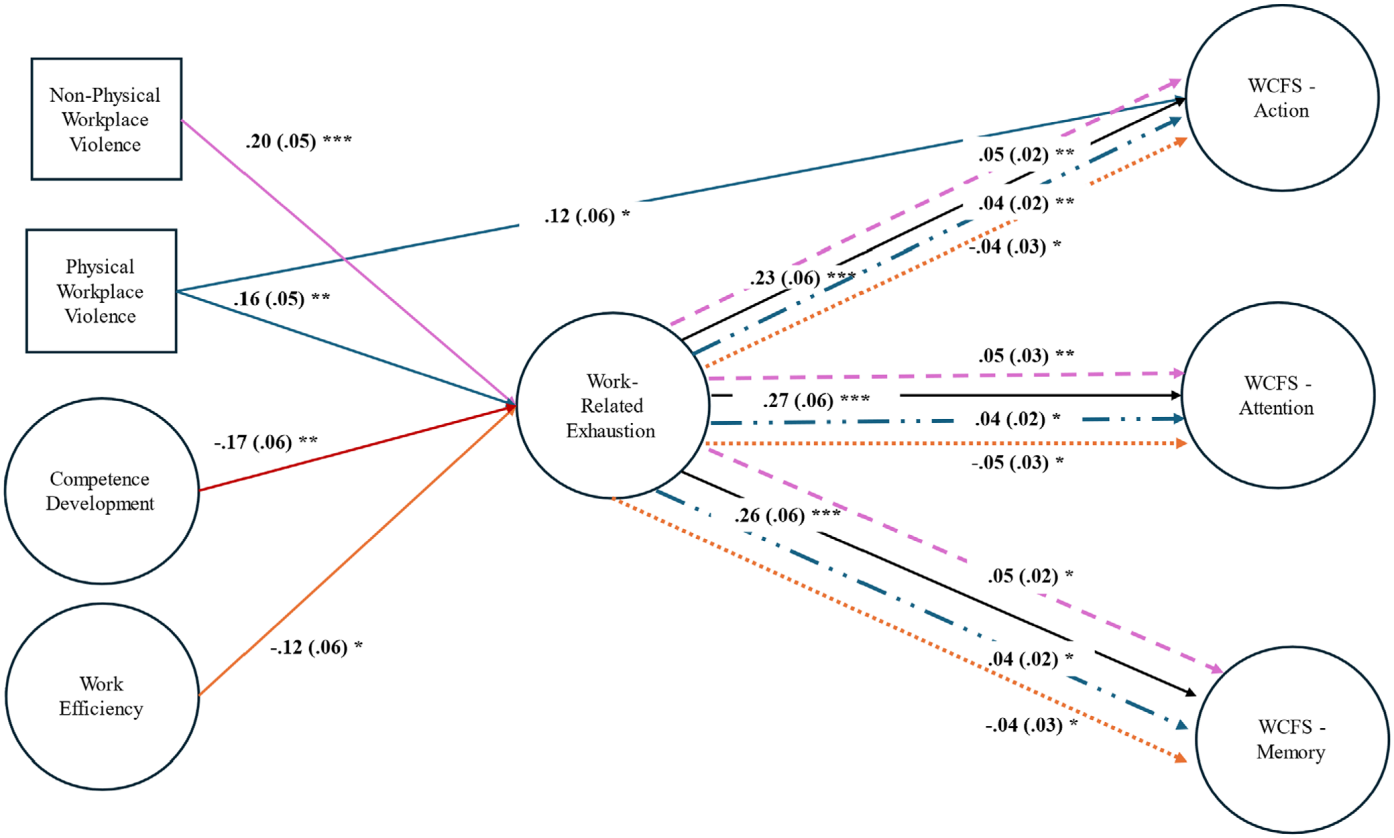
Structural equation model results. Dashed lines reflect indirect effects mediated through work‐related exhaustion. Ovals denote latent variables within the model, standardized error estimates are presented in parenthesis. Only significant associations are illustrated in the model.

Table 4 and Figure 2 display the results of the structural equation modelling. The measurement model fit the data well (*χ* ^2^ = 833.13 (284), CFI = 0.92, RMSEA = 0.07, SRMR = 0.05). The full model also displayed acceptable fit (*χ* ^2^ = 980.51 (374), CFI = 0.91, RMSEA = 0.06, SRMR = 0.06). All paths were estimated in the same model simultaneously. Supporting hypothesis 1, non‐physical violence (*b* = 0.20, *p* < .001, 95% CI [0.10, 0.30]) and physical violence (*b* = 0.16, *p* = .01, 95% CI [0.06, 0.26]) were significant predictors of increased work‐related exhaustion. Non‐physical and physical violence were also examined as direct predictors of the WCFS subscales. Physical violence was only a significant predictor of the action‐related subscale (*b* = 0.12, *p* = .02, 95% CI [0.02, 0.25]), providing only marginal support for hypothesis 2. The hypothesized positive effects of workplace efficiency (*b* = −0.17, *p* = .01, 95% CI [−0.29, −0.05]) and competence development (*b* = −0.12, *p* = .05, 95% CI [−0.23, −0.01]) in predicting lower work‐related exhaustion (hypothesis 4) were also supported. Hypothesis 5 was unsupported, as there were no direct effects of workplace efficiency or competence development on any of WCFS dimensions. In support of hypothesis 7, work‐related exhaustion predicted significantly higher scores for the memory (*b* = 0.26, *p* < .001, 95% CI [0.15, 0.37]), attention (*b* = 0.27, *p* < .001, 95% CI [0.16, 0.38]), and action subscales (*b* = 0.23, *p* < 0.001, 95% CI [0.12, 0.34]). Age was included as a control variable in all analyses, but its effects were only significant in relation to the memory subscale of WCFS (*b* = −0.11, *p* = 0.03, 95% CI [−0.21, −0.01]). Prior COVID‐19 infection was also included as a control variable, but its effects were not significant.

**TABLE 4:** Structural equation modelling results.

Variable	*B*	*SE*	*p*
WRE predictors			
Non‐physical violence exposure	0.2	0.05	<.000***
Physical violence exposure	0.16	0.05	<.01**
Workplace efficiency	−0.17	0.06	<.01**
Competence development	−0.12	0.06	.05*
Memory WCFS predictors			
Non‐physical violence exposure	0.03	0.06	.63
Physical violence exposure	0.08	0.06	.18
Workplace efficiency	−0.1	0.07	.12
Competence development	−0.04	0.06	.5
WRE	0.26	0.06	<.000***
Age	−0.11	0.05	.03*
COVID Positive	−0.03	0.05	.57
Attention WCFS predictors			
Non‐physical violence exposure	0.05	0.06	.63
Physical violence exposure	0.08	0.06	.17
Workplace efficiency	−0.04	0.07	.58
Competence development	−0.06	0.06	.36
WRE	0.27	0.06	<.000***
Age	0.02	0.05	.76
COVID positive	0.08	0.05	.1
Action WCFS predictors			
Non‐physical violence exposure	0.01	0.06	.93
Physical violence exposure	0.12	0.06	.02*
Workplace efficiency	−0.04	0.07	.6
Competence development	−0.04	0.06	.52
WRE	0.23	0.06	<.000***
Age	−0.1	0.05	.07
COVID positive	0.08	0.05	.35
Indirect effects on memory WCFS via WRE			
Non‐physical violence exposure	0.05	0.02	<.01**
Physical violence exposure	0.04	0.02	.01*
Workplace efficiency	−0.04	0.03	.02*
Competence development	−0.03	0.02	.07
Indirect effects on attention WCFS via WRE			
Non‐physical violence exposure	0.05	0.03	<.01**
Physical violence exposure	0.04	0.02	.01*
Workplace efficiency	−0.05	0.03	.02*
Competence development	−0.03	0.02	.07
Indirect effects on action WCFS via WRE			
Non‐physical violence exposure	0.05	0.02	<.01**
Physical violence exposure	0.04	0.02	.02*
Workplace efficiency	−0.04	0.03	.03*
Competence development	−0.03	0.02	.08
Model fit information			
Chi‐square	980.51 (374)		<.000***
RMSEA	0.06		
CFI	0.91		
SRMR	0.06		

*Note*: *N* = 435.

Abbreviations: WCFS, workplace cognitive failure scale; WRE, work‐related exhaustion.

* *p* < .05;

** *p* < .01;

*** *p* < .001.

To test the indirect effects proposed in hypotheses 3 and 6, indirect effects and confidence intervals were estimated using a bias‐corrected bootstrapping approach with 1000 sample draws. The indirect effect of non‐physical violence was significant for WCFS memory (*indirect effect* = 0.05, *p* < .01, 95% CI [0.02, 0.09]), attention (*indirect effect* = 0.05, *p* < .01, 95% CI [0.02, 0.09]), and action (*indirect effect* = .05, *p* < .01, 95% CI [0.01, 0.08]) subscales. Similarly, the indirect effect of physical violence was significant for WCFS memory (*indirect effect* = 0.04, *p* = 0.01, 95% CI [0.01, 0.07]), attention (*indirect effect* = 0.04, *p* < .01, 95% CI [0.01, 0.08]), and action (*indirect effect* = 0.04, *p* = .02, 95% CI [0.01, 0.07]), providing total support for hypothesis 3. Workplace efficiency had significant indirect effects for the WCFS memory (*indirect effect* = −0.04, *p* = .02, 95% CI [−0.08, −0.01]), attention (*indirect effect* = −0.05, *p* = .02, 95% CI [−0.08, −0.01]), and action (*indirect effect* = −0.04, *p* = .02, 95% CI [−0.07, −0.01]) subscales. In contrast, competence development displayed no indirect effects on WCFS subscales, providing partial support for hypothesis 6.

## 6 DISCUSSION

The aim of this study was to examine the relationships between nurses' exposure to workplace violence and self‐reports of workplace cognitive failure. The study also examined whether workplace efficiency and competence development would be protective, i.e., inversely related, to workplace cognitive failure. Central to our conceptual model was work‐related exhaustion, a key aspect of burnout, as a potential mediator of the effects of work‐related factors on workplace cognitive failure. Results revealed that four of our seven hypotheses were fully supported, two were partially supported, and one was rejected.

### 6.1 Prevalence of workplace violence and workplace cognitive failure

Just under 40% of the nurses in this study reported experiencing physical workplace violence in the previous 12 months; 64% reported non‐physical violence. These statistics are comparable to those reported in an online study of U.S. nurses (44% and 68%, respectively) during a 4‐month period in the early phase of the COVID‐19 pandemic (Byon et al., 2022). The current study was conducted post‐pandemic (April 2023), indicating that the prevalence of violence towards U.S. nurses remains high. In a U.S. study conducted 10 years earlier, 29% of hospital employees reported experiencing physical violence and 60% reported non‐physical violence (Arnetz et al., 2018).

A majority of nurses in the current study (74%) endorsed some degree of workplace cognitive failure. This can be compared to 77% in an earlier study of Michigan nurses in 2020 (Arnetz et al., 2021). This high level of endorsement warrants concern, especially considering the impact of work‐related exhaustion and workplace violence on these cognitive issues. Both age and previous COVID infection were included as control variables in the structural equation model, and older nurses reported less memory‐related cognitive failures compared to younger nurses. This might be attributed to a heightened level of experience and routine in nursing tasks. It might also be explained by the “healthy worker effect” (Li & Sung, 1999), i.e., older nurses with memory‐related cognitive issues may have already left the work force. In fact, the literature suggests that turnover in the nursing profession has increased as a result of the recent pandemic (Falatah, 2021; Raso et al., 2021). COVID infection has been attributed to cognitive issues, such as memory problems and brain fog (Nalbandian et al., 2023) but did not seem to influence nurses' reports of cognitive failure in the current study. This suggests that the cognitive failures reported in this study were indeed related to other work exposures, i.e., violence, work‐related exhaustion, and efficiency.

### 6.2 Direct effects of occupational factors on work‐related exhaustion

Violence exposures were positively associated with work‐related exhaustion (H1), while efficiency and competence development were inversely associated (H4). Previous studies of nurses have reported significant associations between workplace violence and burnout (Duan et al., 2019; Kobayashi et al., 2020), although those studies did not examine work‐related exhaustion specifically. A longitudinal study of Danish employees found that work‐related violence at baseline predicted fatigue five years later, independent of covariates such as gender, age, interpersonal conflict, and baseline fatigue (Högh et al., 2003). In a one‐year cross‐lagged study of U.S. hospital employees, work‐related exhaustion was an inverse predictor of work efficiency but the reverse – that efficiency predicted work‐related exhaustion – was not significant (Viotti, Essenmacher, et al., 2018). However, a study of police officers found that exhaustion and work efficacy were mutually related (Taris et al., 2010). In a study of psychotherapists, low ratings of competence predicted higher levels of burnout (Spännargård et al., 2023). These studies offer support for our findings on occupational stressors (violence) and protective factors (efficiency and competence development) as predictors of work‐related exhaustion.

### 6.3 Direct effects of occupational factors on workplace cognitive failure

Our hypotheses regarding the direct effects of occupational factors on the WCFS subscales were largely unsupported. Non‐physical violence did not predict any of the subscales, while physical violence predicted Action, but not the other subscales. Thus, H2 was only partially supported. Previous studies have identified an association between workplace violence exposure and reduced ability to function at work (Ding et al., 2023; Gates et al., 2011; Magnavita et al., 2020). In a study of Chinese nurses, Ding et al. (2023) found that nurses exposed to violence had a higher risk of perceived cognitive deficits. However, a study of Italian nurses found that violence exposure impaired their ability to function at work, but not cognitively (Magnavita et al., 2020). A U.S. study of emergency department nurses reported increased difficulty in staying cognitively focused among nurse victims of violence (Gates et al., 2011). All these studies utilized different measures of cognition and did not specifically measure workplace cognitive failures. In the current study, the significant association between physical violence and the Action subscale, which measures failures such as accidentally dropping objects, throwing something away unintentionally, or activating the wrong machine, is important. These are observable, potentially dangerous actions that could reflect a strong emotional response to the violent event, supporting the notion that violence can be a physically and psychologically traumatic experience (Gates et al., 2011).

As to protective factors, neither work efficiency nor competence development were direct predictors of any of the WCFS subscales, thus rejecting H5. By contrast, a study of Swiss nurses found that work interruptions, a measure of poor work efficiency, were directly associated with the Action subscale (Elfering et al., 2015). A study from Finland found that younger nurses with less experience and skills related to electronic health record use had more cognitive failures than older nurses with better skills (Kaihlanen et al., 2020). However, having the necessary skills to do one's work and having the opportunity to develop one's competence at work, though similar, are not equivalent measures.

### 6.4 Indirect effects of occupational factors on workplace cognitive failure via work‐related exhaustion

Both types of violence (H3) and work efficiency, but not competence development (H6), were significant indirect predictors of the three WCFS subscales via work‐related exhaustion. Thus, H3 was fully supported while H6 was only partially supported. Moreover, work‐related exhaustion was a significant predictor of each of the three subscales, supporting H7. The central importance of work‐related exhaustion, both as a mediator of the work‐related exposures but also as a direct predictor of WCFS subscales, was thus confirmed.

Work‐related exhaustion captures the experience of complete fatigue that can accompany a nurses' work. In a study of nurses working in home care and nursing homes, work‐related exhaustion was the strongest inverse predictor of work satisfaction (Hasson & Arnetz, 2008). Ding et al. (2023) found that nurses who experienced workplace violence reported greater cognitive deficits and had significantly worse mental and emotional health in the form of anxiety, depression, poor sleep, and loneliness. While the current study did not examine mental health per se, the central mediating role played by work‐related exhaustion in cognitive failures is in line with those findings. Moreover, work‐related exhaustion displayed significant direct effects on all three of the cognitive failure subscales, further supporting the findings by Ding et al. (2023). Of note, among all independent variables, work‐related exhaustion was the only significant predictor of each cognitive failure subscale (*p* < .000 on all three), with the exception of the effect of physical violence on the action subscale (*p* < .05).

Efficiency's impact on cognitive failures was only significant via work‐related exhaustion. Previous research has shown a significant correlation between workflow interruptions, a measure of poor work efficiency, and cognitive failure in action regulation (Elfering et al., 2014, 2015). Poor efficiency and cognitive failure were associated with surgical errors (Elfering et al., 2014) and nurses' reports of near accidents (Elfering et al., 2015). In both of those studies, the impact of poor efficiency on patient outcomes was indirect, mediated via cognitive failure of action. While further research is needed to better understand the role of work efficiency in cognitive failure, the current study's findings are in line with this previous work.

Although competence development was significantly correlated with WCFS and each of the subscales in bivariate analyses, it did not show significant effects either directly or indirectly in the structural equation model. Competence development only showed a marginally significant association with work‐related exhaustion (*p* = .05) when included in the model with efficiency and violence; all other associations were non‐significant. It is possible that competence development may contribute to nurses' cognitive stimulation (Marquie et al., 2010) and work satisfaction (Hasson & Arnetz, 2008), but this may not be sufficient to protect against cognitive failure in an environment marked by workplace violence and work‐related exhaustion. In contrast, nurses' experience of workplace violence may trigger a more profound physical, emotional, and cognitive response that influences cognitive failure. Workplace efficiency, the other protective factor, has been shown to enhance employee mental health (Arnetz, Lucas, & Arnetz, 2011) and this may explain its protective effects on cognitive failure via work‐related exhaustion.

The central role played by work‐related exhaustion in this study's findings offers support for the Conservation of Resources theory. While only one of the four independent occupational factors (physical violence) showed direct effects on cognitive failure, all but competence development showed indirect effects through work‐related exhaustion. Workplace violence contributes to work‐related exhaustion, resulting in a resource loss that was associated with greater workplace cognitive failure. Conversely, workplace efficiency was associated with decreased work‐related exhaustion, indicating a resource gain that was protective against cognitive failure.

### 6.5 Strengths and limitations

The study used a cross‐sectional design and causality between work environmental factors and workplace cognitive failure cannot be determined. Nevertheless, the study strength was the use of structural equation modelling (SEM) which enabled the analysis of both direct and indirect linear relationships among variables while accounting for measurement error. SEM enables the investigation of complex relationships through statistical modelling based on hypothesis testing. SEM also provides estimates of relationships among latent variables (measurement model) as well as relationships among constructs (structural model, Tomarken & Waller, 2005). The study utilized a convenience sample and nurses who responded may not be representative of all nurses in Michigan or other geographic areas. However, the sample was similar to the total population of Michigan nurses in terms of gender and ethnicity. The response rate on the study was low (3%) but similar to other online surveys of nurses conducted in recent years (Arnetz, Goetz, Sudan, et al., 2020; Byon et al., 2022; Sagherian et al., 2020). Finally, all measures in the study were self‐reported, and results may have been affected by respondent bias. For instance, social desirability biases may have influenced the degree to which nurses were willing to endorse workplace cognitive failure items (Podsakoff et al., 2003).

### 6.6 Recommendations for further research

Current research on workplace cognitive failure among nurses has utilized a variety of measures, making comparisons of study results difficult. Future research would benefit from a precise definition of cognitive failure and its measurement among healthcare professionals. Future studies focused on workplace cognitive failure would also benefit by the inclusion of administrative data on reported violent incidents and/or adverse events associated with nurses' cognitive failures.

### 6.7 Implications for policy and practice

This study highlights the potential negative impact of violent incidents in the healthcare workplace on nurses' cognitive function. Importantly, the effects of violence on cognitive failure were mostly indirect via work‐related exhaustion. These findings underscore the importance of healthcare organizational efforts to reduce not just violent events but other potential sources of work‐related exhaustion among nurses. Healthcare organizations should encourage nurses to report incidents of workplace violence and provide opportunities for them to review incidents with colleagues as part of a violence management and prevention plan (Arnetz et al., 2017; Hamblin et al., 2017; Joint Commission, 2021). Nurse managers should also raise awareness of signs of workplace cognitive failure and its potential implications for patient safety. Organizational efforts should also be made to enhance workplace efficiency to counteract both work‐related exhaustion and workplace cognitive failure among nurses.

## 7 CONCLUSIONS

Both occupational stressors (workplace violence, work‐related exhaustion) and protective factors (work efficiency) were significant predictors of workplace cognitive failure in a sample of U.S. nurses. Importantly, both violence and efficiency exhibited primarily indirect effects on workplace cognitive failure via work‐related exhaustion. With nurses today reporting increased workplace violence (Byon et al., 2022) and extremely high rates of burnout (Aiken et al., 2023), the central role of work‐related exhaustion, an indicator of burnout, in this study has important implications. Results suggest that efforts to prevent workplace violence and work‐related exhaustion, and enhance work efficiency, may help to mitigate cognitive failure. Interventions to prevent and mitigate work‐related exhaustion may also help nurses counteract the cognitive effects of violence exposure. As nurses continue to face tremendous work strain in the post‐pandemic era, it is important to safeguard them from workplace cognitive failure, which can negatively impact nurses' well‐being and patient safety.

## FUNDING INFORMATION

This research did not receive any specific grant from funding agencies in the public, commercial or not‐for‐profit sectors.

## CONFLICT OF INTEREST STATEMENT

No conflict of interest has been declared by the authors.

### PEER REVIEW

The peer review history for this article is available at https://www.webofscience.com/api/gateway/wos/peer‐review/10.1111/jan.16217.

## Data Availability

The data that support the findings of this study are available from the corresponding author upon reasonable request.
